# Natural variation and genetic loci underlying resistance to grain shattering in standing crop of modern wheat

**DOI:** 10.1007/s00438-023-02051-z

**Published:** 2023-07-06

**Authors:** Livinus Emebiri, Shane Hildebrand

**Affiliations:** NSW Department of Primary Industries, Wagga Wagga Agricultural Institute, Wagga Wagga, NSW 2650 Australia

**Keywords:** Wheat, *Triticum aestivum*, Grain shattering, Genetic mapping, Quantitative trait locus

## Abstract

Modern wheat (*Triticum aestivum* L.) cultivars have a free-threshing habit, which allows for easy manual or mechanical threshing. However, when harvesting is delayed or extreme weather events occur at harvest time, grain shattering can cause severe loss of harvestable yield. In the past, grain size was considered a predisposing factor as large, plump kernels can lead to buckling and breaking of the outer glume, but the correlation between glume strength and shattering is not strong in modern wheat, and it is hypothesised that there may be other genetic mechanisms. Data from two bi-parent populations and a wheat diversity panel were analyzed to explore the underlying genetic basis for grain shattering observed in multiple field experiments through quantitative trait loci (QTL) analysis. Grain shattering had a significant and negative association with grain yield, irrespective of populations and environments. The correlation with plant height was positive in all populations, but correlations with phenology were population specific, being negative in the diversity panel and the Drysdale × Waagan population, and positive in the Crusader × RT812 population. In the wheat diversity panel, allelic variations at well-known major genes (*Rht-B1, Rht-D1* and *Ppd-D1*) showed minimal association with grain shattering. Instead, the genome-wide analysis identified a single locus on chromosome 2DS, which explained 50% of the phenotypic variation, and mapping to ~ 10 Mb from *Tenacious glume* (*Tg*) gene. In the Drysdale × Waagan cross, however, the *reduced height* (*Rht*) genes showed major effects on grain shattering. At the *Rht-B1* locus, the *Rht-B1b* allele was associated with 10.4 cm shorter plant height, and 18% decreased grain shattering, whereas *Rht-D1b* reduced plant height by 11.4 cm and reduced grain shattering by 20%. Ten QTL were detected in the Crusader × RT812, including a major locus detected on the long arm of chromosome 5A. All the QTL identified in this population were non-pleiotropic, as they were still significant even after removing the influence of plant height. In conclusion, these results indicated a complex genetic system for grain shattering in modern wheat, which varied with genetic background, involved pleiotropic as well as independent gene actions, and which might be different from shattering in wild wheat species caused by major domestication genes. The influence of *Rht* genes was confirmed, and this provides valuable information in breeding crops of the future. Further, the SNP marker close to *Tg* on chromosome 2DS should be considered for utility in marker-assisted selection.

**Supplementary Information:**

The online version contains supplementary material available at 10.1007/s00438-023-02051-z.

## Introduction

**Fig. 1:**
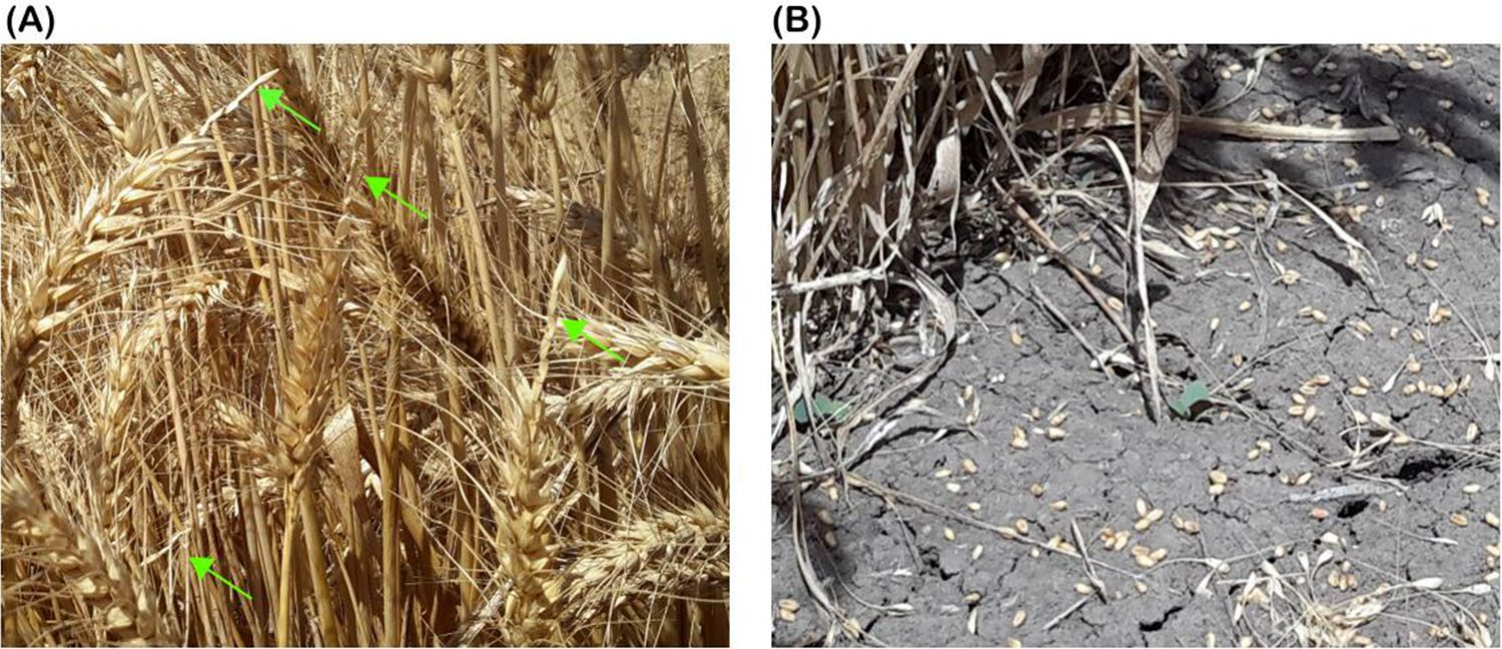
Grain shattering in standing crop of wheat in the field. **A** Grain shattering occurred mostly in the apical spikelet positions on the spike (green arrows), **B** Shattered grains on the ground, which are a direct loss of income to the grower

Modern wheat (*Triticum aestivum* L.) cultivars have a free-threshing habit, with soft glumes that allow for manual or mechanical harvesting. However, when harvesting is delayed or extreme weather events occur at harvest time, grain shattering can account for up to 17% loss of harvestable yield. The propensity to shatter was eliminated in early forms of the domesticated crops (Dubcovsky and Dvorak 2007), but not completely (Li et al. 2006). In the wild type, the head fractures easily at the junction of the rachilla with the rachis but modern wheat have a tough rachis governed by major genes located on homoeologous regions on the short arms of chromosomes 3A (*Br*_*2*_), 3B (*Br*_*3*_) and 3D (*Br*_*1*_) (Nalam et al. 2006; Watanabe et al. 2006). Independent recessive mutations in each of the brittle rachis genes cause the cell wall at specific ‘‘constriction grooves’’ or fracture zones to thicken, converting the wild-type brittle rachis into a tough, non-brittle form that promotes head retention (Pourkheirandish et al. 2015). The brittle rachis, like other domestication traits, is easily distinguishable due to its high penetrance and is heavily selected against during the early generations of breeding programs. Despite heavy selection, however, grain loss due to shattering still occurs in the field in modern wheat (Chang 1943; Clarke and De Pauw 1983). Grain loss in this context is defined as the dropping of individual grains from the rachis at apical end of the spikes (Beck 1951; Porter 1959; Clarke 1981) (Fig. 1a), or the loss of a spike segment and the grains (Kandemir et al. 2000), which is different from the loss of whole spikes, as in barley (Clarke 1981). There are suggestions in the literature that the size of individual grains might be a predisposing factor (Platt and Wells 1949; Clarke 1981) as large, plump kernels and more grains per head can lead to buckling and breaking of the outer glume (bracts enclosing the grain), making the grain more easily removable from the spike (Vogel 1938). Genes that control soft glume (*sog*) and glume tenacity (*Tg*) have been identified and localised to chromosomes 2AS and 2DS (Sood et al. 2009), but according to Zhang et al. (2009), the correlation between glume strength and shattering is not strong in modern wheat. It was hypothesised, therefore, that there may be other genetic mechanisms controlling differences in grain shattering between varieties (Doebley et al. 2006).

Wheat is the second most-widely cultivated crop grown in the world, driven by the plethora of products that can be made from the grain, each of which has a near-limitless number of variations (Kiszonas and Morris 2018). It is pivotal to global food security, with demand now outstripping supply, as global production volume in 2020–2021 of over 700 million metric tonnes was outstripped by the consumption volume of over 710 million metric tonnes (Shahbandeh 2022). It is expected that climate change will exacerbate food security in areas that already currently have a high prevalence of hunger and malnutrition (Wheeler and Braun 2013). Globally, annual wheat yield ranges from < 1 t/ha/year when water or nutrients are limiting to > 10 t/ha/year in cooler, well-watered environments (Asseng et al. 2020). Causes of the yield gap are well documented (see Hochman and Horan 2018) but a significant component that is often overlooked is the loss of harvests in standing crops due to various reasons, including grain shattering.

Grain loss due to shattering (Fig. 1b) is a direct loss of income to the grower, as the more grains a grower can get into the machine at harvest, the greater the returns (Hofman and Kucera 1978). Clarke and De Pauw (1983) calculated the potential loss to range from 3 to 17% of harvestable yield, while Vogel (1938) reported it to be between 5 and 15%. Not only is the grain lost when shattering occurs, but a high amount of volunteer crop can also be expected during fallow, and if uncontrolled, can reduce water storage and contribute to the field seed bank. The amount of grain loss due to shattering depends on environmental conditions, such as high temperatures and wind speed, two weather factors that are predicted to increase in intensity due to climate change. Efforts to limit the loss are focused on cultivar choice and management, and a good understanding of the genetics would enable identifying susceptible genotypes before field trials and allow agronomic research to focus on managing the risk by varietal selection. The objectives of this study were to determine the underlying genetic basis for grain shattering in standing crop of modern wheat through quantitative trait loci (QTL) analysis and investigate the linkage with important agronomic characters.

## Materials and methods

### Diversity panel

The diversity panel comprised of 295 hexaploid and tetraploid genotypes, as well as advanced breeding lines from the International Maize and Wheat Improvement Center (CIMMYT), and landrace cultivars sourced from heat-prone environments. They formed part of a larger research on wheat heat tolerance reported by Collins et al. (2017). Molecular data for the diversity panel were derived from publicly available Illumina iSelect 90 K SNP array markers, made available by Garcia et al. (2019) for more than 500 diverse wheat accessions. The total marker set of 30,548 SNPs was filtered to remove markers with minor allele frequency of 5% or below. Then, the data were further reduced to retain 10,826 markers using PLINK’s function for SNP pruning based on linkage disequilibrium (Purcell et al. 2007). Missing data were imputed using the Random Forest regression method in R package (Stekhoven and Bühlmann 2011).

### *Drysdale* × *Waagan*

This is a population of 142 doubled haploid (DH) lines derived from the F_1_ of a cross between the Australian wheat cultivars, ‘Drysdale’ and ‘Waagan’. Drysdale (named after the Australian artist, Russell Drysdale) was released in 2002 with the pedigree Hartog*3/Quarrion and was one of two varieties (the second was Rees) bred for increased water use efficiency by selecting for reduced carbon isotope discrimination (Richards 2006). Waagan was derived from a cross of Janz with a CIMMYT line, 24IBWSN-244. The point mutations responsible for the two major semi-dwarfing genes *Rht-B1* (Syn. *Rht1*) and *Rht-D1* (Syn. *Rht2*), segregate in the population, with Drysdale carrying the tall allele at *Rht-B1* locus (*Rht-B1a*) and the dwarfing allele (*Rht-D1b*) at *Rht-D1*, and vice versa for Waagan (Shirdelmoghanloo et al. 2016).

Details of the Drysdale × Waagan DH population development and genotyping with the 9 k SNP array (Cavanagh et al. 2013) were described by (Shirdelmoghanloo et al. 2016). The total marker set consisted of 2,711 SNP markers, and these were assigned to physical positions on the wheat genome by searching against the T3/Wheat repository (https://wheat.triticeaetoolbox.org/). From these, a subset of 908 markers located on the wheat genome was selected for QTL analysis. They covered 13.7 Gigabase (Gb) of the 17-gigabase hexaploid bread wheat genome (80.6%), with an average inter-marker interval of 15.1 Mb.

### *Crusader* × *RT812*

The Crusader × RT812 population is a DH population constructed from the F_1_ of a cross between the Australian cultivar, ‘Longreach-Crusader’ (Sunbrook/H45), and a breeding line, ‘RT812’, developed at CIMMYT. RT812 has the pedigree, Pastor//HXL7573/2*Bau/3/CMH82.575/CMH82.801. Crusader is extremely well adapted to main and later season sowings in New South Wales and Queensland, Australia. The population comprised of 243 lines, genotyped with 9,792 DArTseq single-nucleotide polymorphic markers assayed across the whole genome. DArTseq sequences (available at https://www.diversityarrays.com/) were used to query the IWGSC RefSeq v2.1 wheat reference genome for physical positions (Mb) of the markers. Missing values were imputed using the ‘fill.geno” in R/QTL (Broman et al. 2003).

### Field experiments

Eleven field experiments were conducted between 2015 and 2018, and the locations for individual populations are indicated on Table 1. The soil at Wagga Wagga (latitude 35.05° S, longitude 147.35° E) is a sandy clay loam described as a Red Kandosol. It is moderately permeable, moderately well-drained with a greyish brown colour. At Condobolin (latitude 33.07° S, longitude 147.26° E), the soil is red brown in colour with near neutral pH and low inherent fertility and organic matter, and at Leeton (latitude 34.36° S, longitude 146.21° E), the soil is a vertosol, with shrink-swell properties that exhibit strong cracking when dry.

**Table 1:** Overview of considered genetic materials, field experiments, layout, and trait heritabilities in field experiments conducted between 2015 and 2018

Population/Site/year	No. lines	Field layout (C × R)	Heritability estimates				
			Grain shattering	Plant height	Phenology	Grain weight	Grain yield
Drysdale × Waagan							
Wagga Wagga, 2015 Early	144	12 × 18	0.30	0.27	0.39	0.95	0.82
Wagga Wagga, 2015 Late	144	12 × 18	0.85	0.97	0.82	0.92	0.90
Crusader × RT812							
Condobolin, 2017 Early	243	18 × 10	0.79	0.57	0.52	0.91	0.86
Condobolin, 2017 late	243	18 × 10	0.87	0.50	0.86	0.91	0.48
Condobolin, 2018 Early	262	18 × 20	0.42	0.23	0.88	0.85	0.56
Diversity panel							
Elite lines, Leeton, 2015, Early	224	21 × 20	0.83	0.80	0.93	0.96	0.82
Elite lines, Leeton, 2015, Late	224	21 × 20	0.68	0.92	0.93	0.96	0.90
Elite lines, Wagga Wagga, 2015, Early	231	23 × 18	0.85	0.91	0.78	–	0.85
Elite lines, Wagga Wagga, 2015, Late	231	18 × 23	0.76	0.82	0.79	–	0.82
Landraces, Leeton, 2015, Late	64	6 × 20	0.65	0.80	–	–	0.85
Landraces, Wagga Wagga, 2015, Late	64	7 × 18	0.70	0.71	–	–	0.91

Field layout refers to the number of columns (C) and rows (R) used to ensure genotype allocation would be spatially balanced across the experiment. Heritability estimates represent the fraction of total variation due to genetic factors

The experiments were sown with entries in a partial replication (p-rep), in which 75% of the entries were duplicated twice. Plot size was 7.5 m^2^ (6 rows with 30 cm spacing, 6 m long), and sown with 60 g seed. All experiments were fertilized at the time of sowing with mono-ammonium phosphate (MAP) at the rate of 100 kg ha^−1^. Weed and disease control, including irrigation, followed standard procedures described in Sissons et al. (2018).

Resistance to shattering was based on visual scoring using a scale of 1 to 9, in which 1 = no shattering and 9 = severe shattering (Haley et al. 2005). Plant height was measured in each plot, from soil surface to tip of the spike and defined as the average of three locations within each plot. Phenology was measured as the date of awn emergence, defined in days from sowing to when awns on 50% of the plants in a plot were approximately 1 cm above the flag leaf auricle (Sissons et al. 2018).

Plots were trimmed from 6 m long to 5 m prior to harvest, and grain yields were harvested using a plot machine, and converted to tonnes per hectare, based on the weight of uncleaned seed from each plot. A sub-sample of 300 g grains were taken from each plot and used to determine grain size, first by counting out 250 kernels randomly on a grain counter (Numigral, Rousseau, Paris, France), and then weighing the kernels, and the weight expressed as the weight of 1000 kernels.

### Statistical analysis

Statistical analyses were performed in R (https://cran.r-project.org/) using mixed linear models to partition the phenotypic variance into genetic and non-genetic components. A baseline model was fitted within a multi-environment framework, which involved using information of the plot layouts (rows and columns) to model position of each plot. The analyses were performed using ASREML-R (version 4) package (Butler et al. 2017) according to the following form:

1 $$y=X\beta +{\mathrm{\rm Z}}_{g}+\varepsilon$$

where *y* is a vector of trait observations, β is a vector of fixed effects to test for trends in the row and column directions, *g* is a vector of the underlying genetic variation of the trait among the progeny lines, *X* and *Z* are the associated design matrices, and ε is a vector of random residual errors. The residual variance also included a correlation structure parameterized as AR1 × AR1 (AR1 = autoregressive first order process) to model the correlation along the rows and columns due to neighbourliness of the experimental plots. These were fitted separately for each environment as fixed factors using the ASReml-R function *at()*, tested for significance (*P* < 0.05) using the wald statistics, and non-significant terms were removed from the final model. Normality checking was performed on the residuals as suggested in Kozak and Piepho (2018), and phenotypes with extreme values were replaced with ‘NA’ in multiple iterations until no residuals beyond ± 3 standard deviations were left.

Heritability (*h*^*2*^) was calculated as described by Cullis et al. (2006):

$$h.2\hspace{0.17em}=\hspace{0.17em}1-\frac{{(mean.sed)}^{2}}{2{\sigma }_{g}^{2}}$$

where *mean.sed* is the average pairwise prediction error of the genetic effects for the tested genotypes and σ^2^g is the genetic variance for the respective treatments.

Genetic correlations (*r*_A_) between grain yield, grain shattering and agronomic traits were calculated as:

$$r\mathrm{A }=\mathrm{ Cov}xy/\sqrt{{\sigma }_{x}^{2}{\sigma }_{y}^{2}}$$

where σ^2^_*x*_ and σ^2^_*y*_ are the genotypic variance components for traits *x* and *y*, and Cov_*xy*_ is the genetic covariance component, calculated after creating a dummy variable (*z* = *x* + *y*) as [(σ^2^_z_ − (σ^2^_*x*_ + σ^2^_*y*_)]/2. The statistical significance of *r*_A_ was assessed as proposed by Schefler (1979), assuming a population correlation coefficient of zero, as:*t* = $\frac{{r}_{A}}{\sqrt{(1-{r}_{A}^{2})/(n-2)}}$where *t* is a student’s *t* value with (*n*-2) degrees of freedom, and n is the number of pairs of observations.

### Genome-wide association analysis (GWA)

The population used for GWA comprised of 131 genotypes selected from the diversity panel for their ploidy (2*n* = 6*x* = 42) and availability of high-density molecular data, including KASP markers for the presence of well-known major semi-dwarf genes, *Rht-B1* and *Rht-D1*, and photoperiod response gene, *Ppd-D1*. Population structure in the GWA panel was analyzed using discriminant analysis of principal components (DAPC) in the adegenet R package (Jombart et al. 2010). The final marker matrix after pre-processing contained 131 individuals and 3,711 markers. Genome-wide association (GWA) analysis was performed with the R package, GAPIT3 (Lipka et al. 2012), using the multi-locus mixed model approach (mlmm) described by Segura et al. (2012), which accounts for kinship. The method uses forward–backward stepwise regression to sequentially incorporate significant markers as covariates in the GWA model before scanning all other markers. Four principal component dimensions, fitted as fixed effects, were used to control for the confounding effect of population structure, along with a kinship matrix calculated according to VanRaden (2008) to define the variance and covariance structure of random individual effects (Yu et al. 2006). Markers exhibiting a genome-wide adjusted FDR *P* value < 0.05 were identified as significant.

### Bi-parent QTL analysis

QTL analysis was performed using the Whole Genome Average Interval Mapping (RWGAIM) algorithm (Verbyla et al. 2012). The SNP molecular marker data were imported using the ‘read.cross’ command of R/QTL (Broman et al. 2003), and converted into an ‘interval’ object using the ‘cross2int’ command of R/WGAIM, with co-located markers placed in consensus bins. Then, the genetic ‘interval’ datum was merged with the baseline model defined in (1), and in the final step, R/WGAIM extended the model by incorporating all markers simultaneously as random covariates to detect main effect QTL. In the R script, the ‘fix.lines’ command was set to TRUE to fix the lines that do not exist in the genetic map. The gen.type = "marker" and the genome-wide Type I error for declaring a significant QTL was set at 0.01. Detailed summaries of detected QTL, including their position, effect size, and level of significance were provided by the package, and the QTL graphically displayed using linkMap() function.

### Pleiotropic QTL analysis

In this step, we performed conditional genome scans using plant height as a fixed covariate in the R/WGAIM analysis of grain shattering. Comparison of unconditioned and conditioned scans would reveal changes in the LOD score for a QTL (Li et al. 2006), and if an unconditioned QTL is still detected after removing the influence of plant height, then this would likely represent an independent locus, but if the QTL is no longer significant, then that would suggest a pleiotropic QTL, causally linked to grain shattering through the effect of plant height. This approached yielded results that were identical to those from conditional genetic analysis proposed by Zhu (1995) and in some cases, revealed new QTL whose presence was made possible by removing the influence of plant height.

### *QTL* × *E analysis*

Following the approach used by many authors (e.g., Lukens and Doebley 1999; Jermstad et al. 2003; Weinig et al. 2003; Geshnizjani et al. 2020), we used analysis of variance (ANOVA) to test the QTLs for environmental sensitivity. The approach also served to validate results obtain by RWGAIM algorithm.

### Bioinformatic analysis

The physical positions of all SNP markers were determined by aligning the sequences harbouring each SNP to the updated reference sequence of the wheat genome (RefSeq v2.1) by BLAST search through the URGI portal (https://wheat-urgi.versailles.inra.fr/). The start position of each locus was extracted from the BLASTN output and used in the QTL analyses.

## Results

### Genotypic variation in landrace and elite wheat

**Fig. 2:**
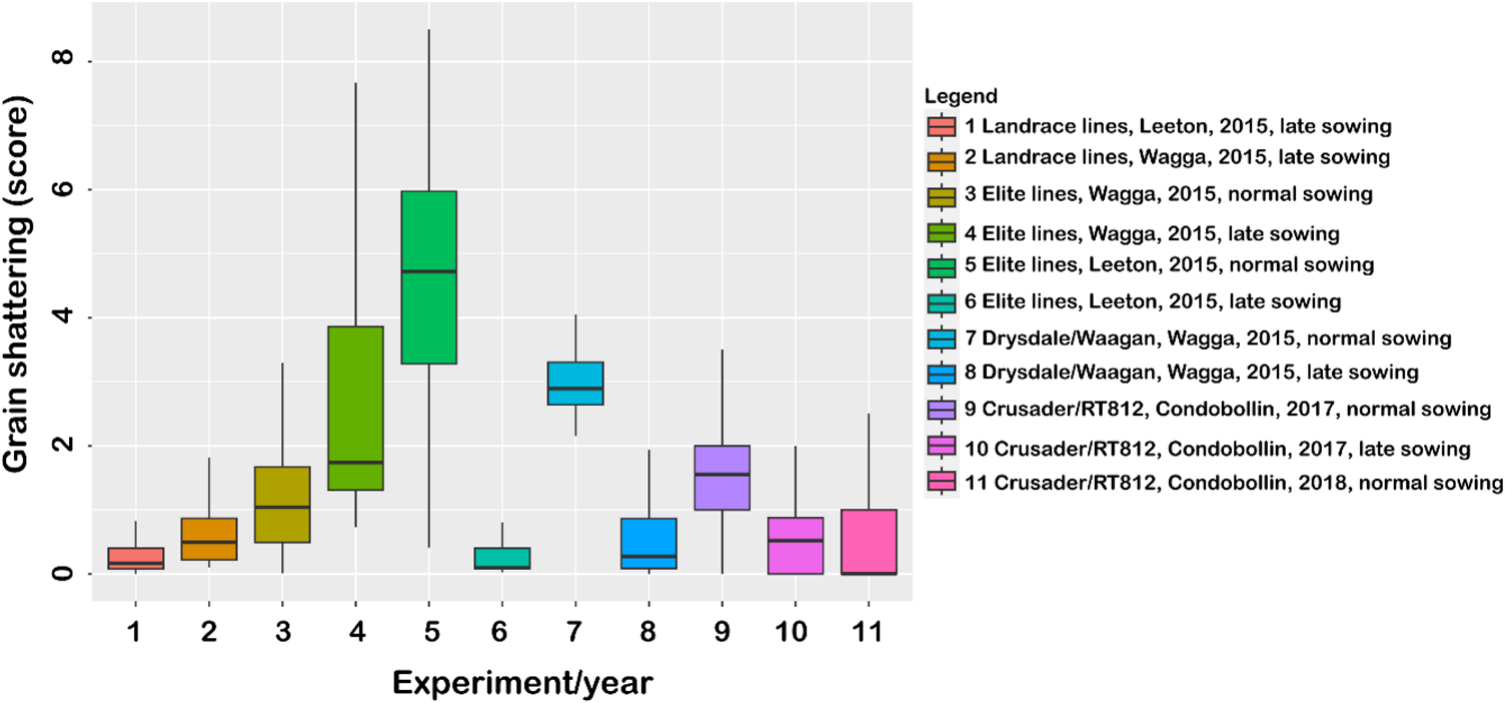
Observed phenotypic variation for grain shattering in standing crop of multiple wheat populations grown in different field experiments between 2015 and 2018

**Fig. 3:**
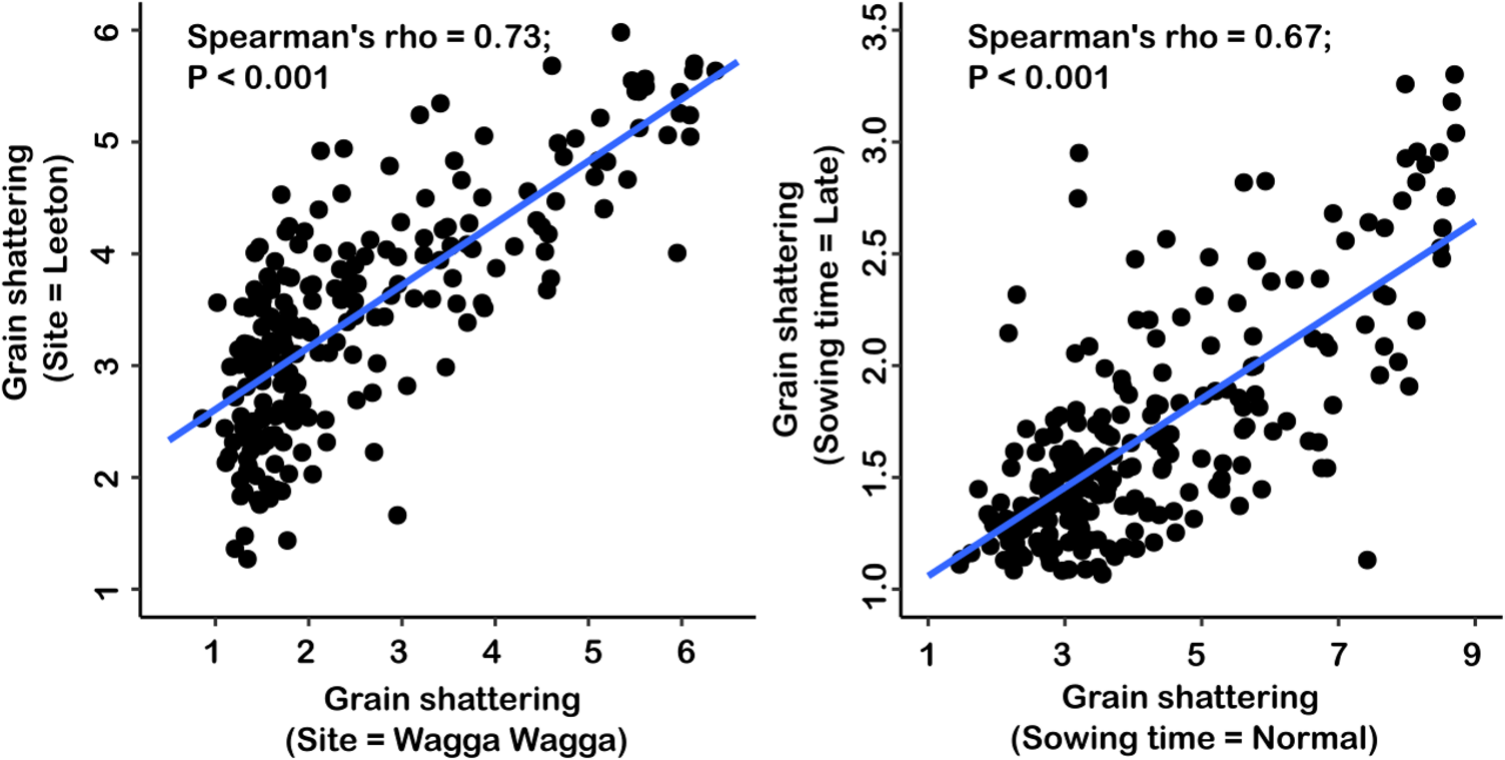
Scatterplot of grain shattering measured in elite wheat cultivars grown at two sites (**a**) and sowing times (**b**) in the wheat diversity panel

Eleven field experiments were monitored for grain shattering in 2015, 2017 and 2018, and significant genotypic variations were observed in all cases (Fig. 2; Table 1). The variability in grain shattering was largest in the elite wheat cultivars grown under normal (June sowing) conditions, as opposed to those grown under late (August) sowing conditions (Fig. 2). Broad-sense heritability estimates obtained for the different population/year data (Table 1) showed higher genetic variances relative to error in eight of the eleven field experiments. This indicates high trait repeatability, which ranged from 0.30 to 0.87. Equally important, these values compared well with those for plant height (Table 1), suggesting either a strong, independent genetic basis for the varietal differences in grain shattering, or pleiotropic effect of genes controlling plant height. Using data for the elite wheat cultivars (because of the diverse genetic background), we found grain shattering in standing crop to show a high degree of repeatability across environments (Fig. 3), with highly significant positive correlation across sites (*r* = 0.77; *P* < 0.001) and time of sowing (*r* = 0.73; *P* < 0.001).

### Genetic correlations

Grain shattering had a negative association with grain yield, irrespective of populations and environments (Table 2). The degree of association was small (− 0.07) in only 1 of the 11 population/year experiments we analyzed and was strong and consistently significant in majority of the experiments, ranging from – 0.20 to – 0.83 (Table 2). The correlations with grain size were positive, indicating that large grains increased the propensity to shattering, but data were not complete, particularly in the landraces, which were compromised by heavy lodging and had to be discarded.

**Table 2:** Genetic correlation coefficients^†^ of grain yield with shattering and other agronomic traits in different wheat populations averaged across sites and years

Population/site/year	Grain shattering vs:			
	Grain yield	Plant height	Phenology	Grain size
Drysdale × Waagan				
Wagga Wagga, 2015 Early	− 0.07 ns	0.25**	0.07 ns	0.09 ns
Wagga Wagga, 2015 Late	− 0.50***	0.67***	− 0.32***	0.06 ns
Crusader × RT812				
Condobolin, 2017 Early	− 0.69***	0.14*	0.09 ns	0.11 ns
Condobolin, 2017 late	− 0.75***	0.25**	0.17**	0.12 ns
Condobolin, 2018 Early	− 0.83***	0.08 ns	0.37***	0.26***
Diversity panel				
Elite lines, Leeton, 2015, Early	− 0.36***	0.32***	− 0.30***	0.25***
Elite lines, Leeton, 2015, Late	− 0.11 ns	0.37***	− 0.30***	0.40***
Elite lines, Wagga Wagga, 2015, Early	− 0.43***	0.38***	− 0.19**	–
Elite lines, Wagga Wagga, 2015, Late	− 0.20**	0.41***	− 0.05 ns	–
Landraces, Leeton, 2015, Late	− 0.50***	0.12 ns	–	–
Landraces, Wagga Wagga, 2015, Late	− 0.29*	0.18 ns	–	–

Signif. codes: < 0.001 '***'; 0.01 '**'; '*' 0.05; ns = Not significant, ‘- ‘ = Not available

^†^A genetic correlation coefficient measures the degree of association between the genetic variations of two quantitative characters in a population (Reeve 1955)

The correlation with plant height was positive in all populations, and strongest in the Drysdale × Waagan doubled haploids, which segregated for the semi-dwarfing genes. The genetic correlation with plant height was also strong in the diversity panel, which comprised of genotypes with different genetic background, but was weak in the Crusader × RT812 population. Phenology had a significant effect on the propensity to grain shattering, but the influence was population-specific (Table 2), being negative in the diversity panel and the Drysdale × Waagan populations, and positive in the Crusader × RT812 population.

### Genome-wide study of diversity panel

**Fig. 4:**
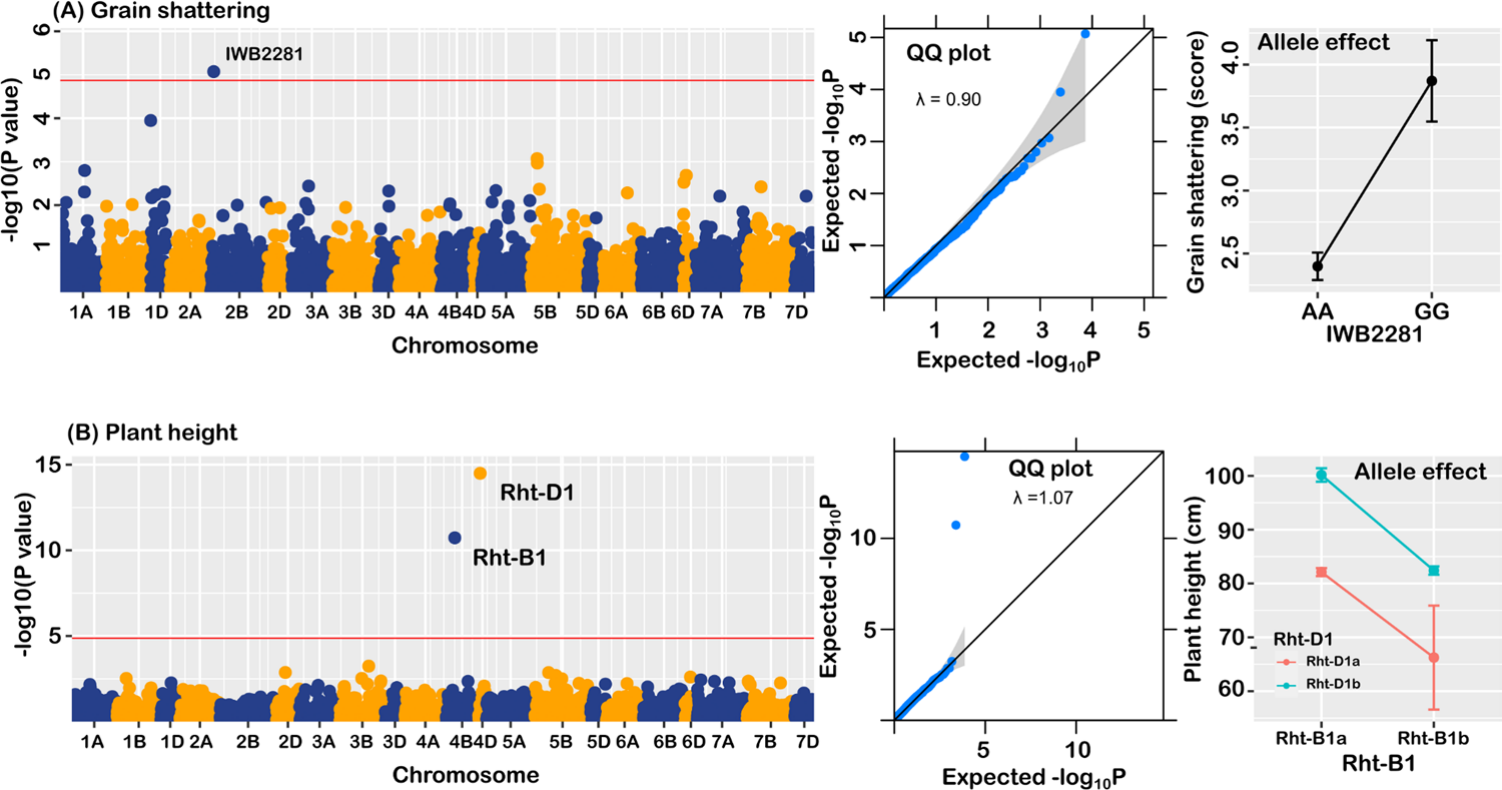
Summary plot of GWA results in a wheat diversity panel, showing **A** Manhattan plot, QQ plot, and allele effect of identified QTL on grain shattering, **B** Manhattan plot, QQ plot, and allele effect of major genes present for plant height in the diversity panel

The GWA identified a marker (Fig. 4), BobWhite_c2949_1083 (Syn. IWB2281), which explained 50% of the phenotypic variability (GAPIT estimate). This could be due to the low statistical power associated with the relatively small population size, but to confirm the GWA result was not spurious, we analyzed data on plant height in the panel, which was genotyped with KASP markers that targeted major semi-dwarf genes, *Rht-B1* and *Rht-D1*. The analysis identified the presence of the major genes in the panel, and their allelic effects conformed to expectations (Fig. 4).

For grain shattering, the quantile–quantile (QQ) plot (Fig. 4) showed further evidence that observed *P* values closely adhered to the expected values, with the genomic inflation factor less than 1.0 (λ = 0.90), indicating there were no systematic, spurious associations due to confounding factors. In the consensus wheat 90 K SNP array (Wang et al. 2014), the identified marker, BobWhite_c2949_1083 (Syn. IWB2281), was placed on chromosome 2BS, but physical mapping localised the SNP marker to the short arm of chromosome 2D, based on the recent Chinese Spring genome assembly (RefSeq v2.1).

### *QTL detection in Drysdale* × *Waagan*

Both Drysdale and Waagan were present in the diversity panel, and they carried the alternate, non-shattering allele identified for grain shattering in the GWA analysis. In field experiments, however, Waagan ranked better than Drysdale for grain shattering, and their doubled haploid progenies were significantly different, with moderate-to-high heritability observed across the sowing times, and estimated at an average of 57.6% (Table 1). For plant height, the average heritability was 62.0%, and for phenology, it was 60.4%. The comparable heritability values showed grain shattering to be under strong genetic control, and further investigations were undertaken to unravel the genetic basis by scanning the wheat genome for allelic differences associated with the phenotype.

**Fig. 5:**
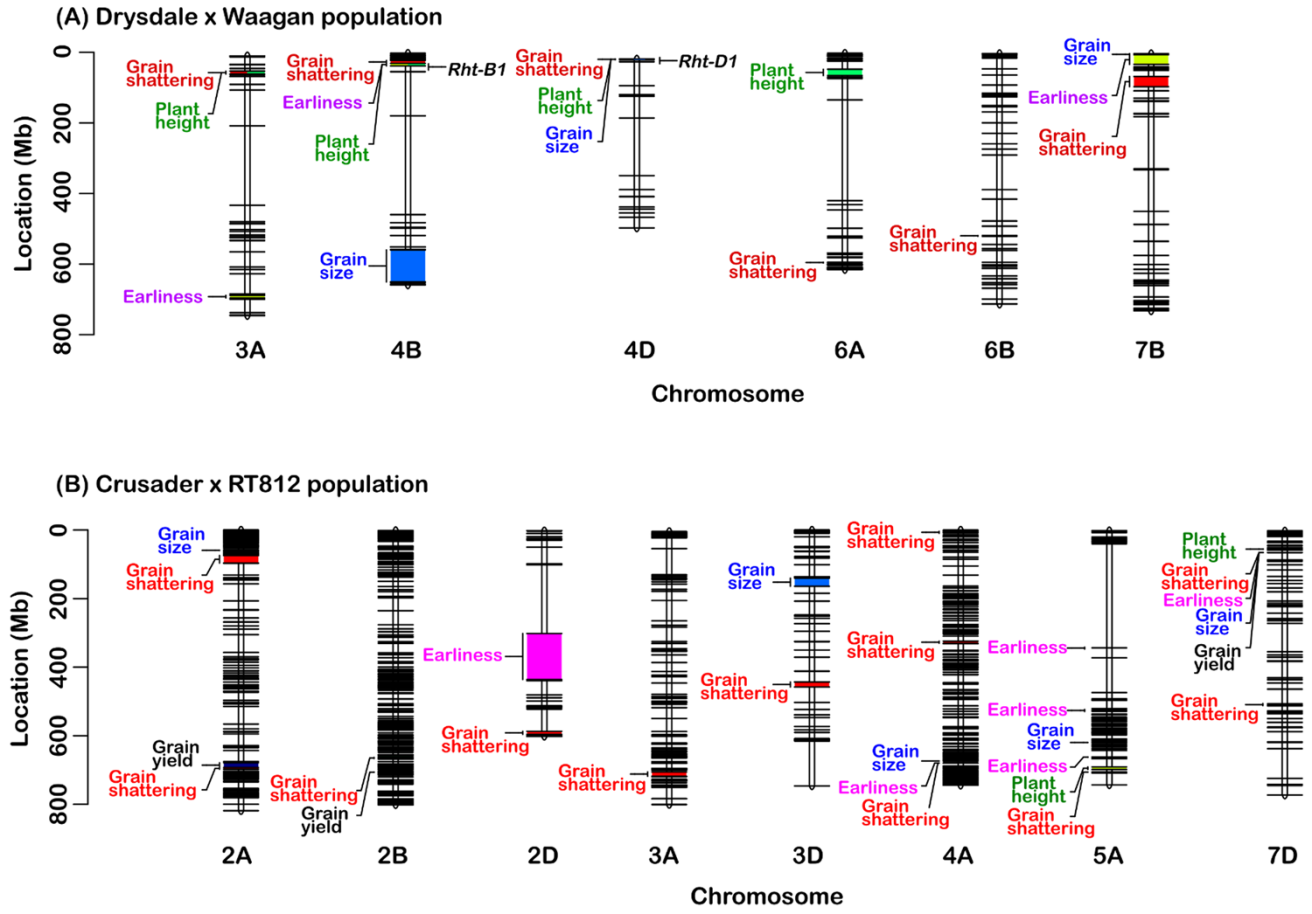
Chromosomal location of main-effect QTL identified for grain shattering and agronomic traits in two bi-parent populations of elite wheat cultivars. Chromosomes harbouring the QTL are represented by vertical lines, and each horizontal black line represents one of the unique SNP markers. Physical distances are reported on the scales to the left of the chromosomes

**Fig. 6:**
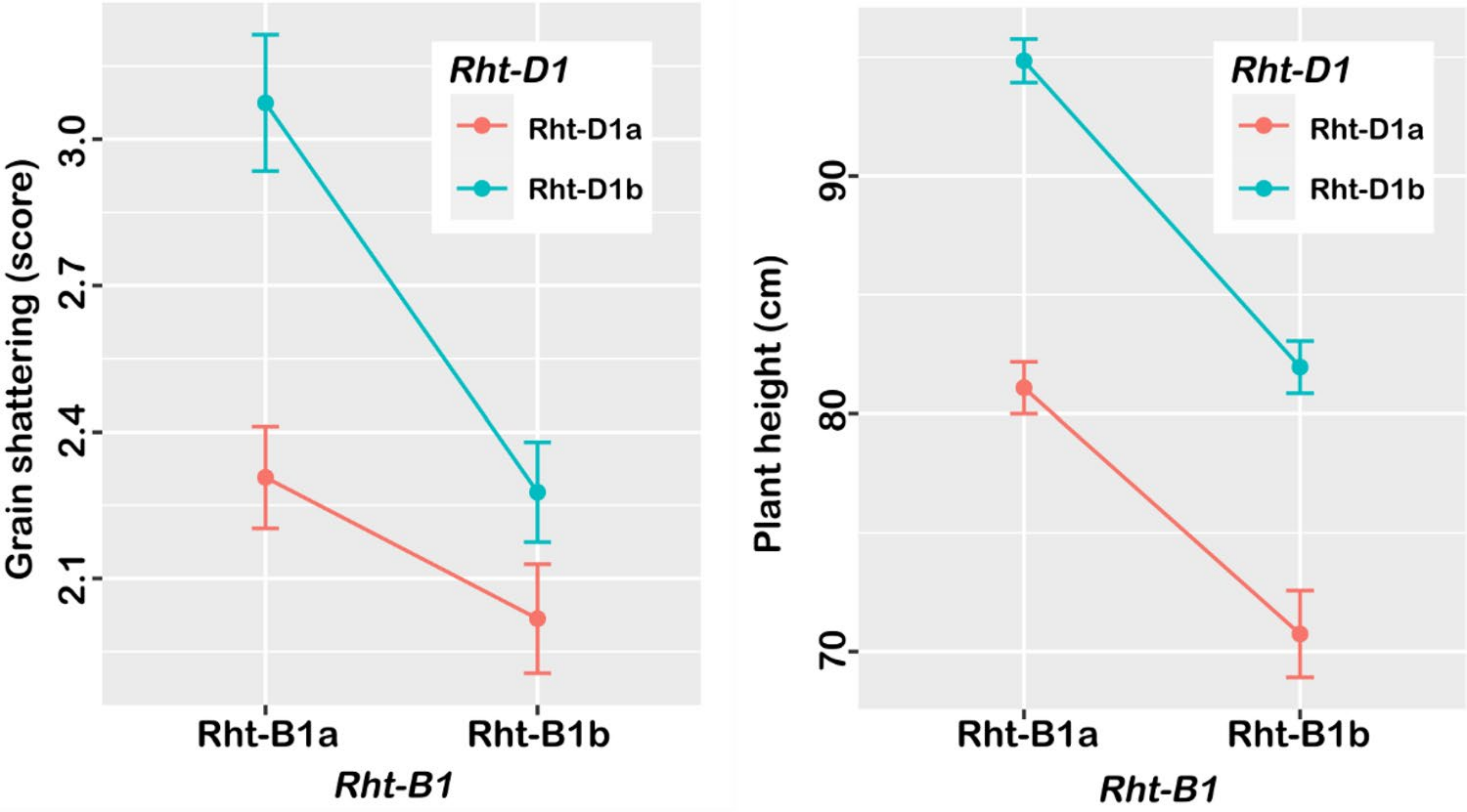
Allelic effects of the major *Rht* gene loci on grain shattering and plant height in the Drysdale × Waagan bi-parent population

In the R/WGAIM analysis, 857 SNP markers representing unique, non-redundant marker bins were used for QTL analysis. The markers satisfied the expected ratio of 1:1 segregation, with 50.9% of ‘AA’ alleles, and 49.1% of the ‘BB’ alleles. Six genomic regions were found to be significantly linked to the variability in the grain shattering (Fig. 5a), and all QTL were verified to be significant (P < 0.01) by independent ANOVA tests (Table 3). Two of the QTL had major effects, collectively explaining almost 50% of the phenotypic variation. The two major QTL were located on chromosomes 4B and 4D, and directly linked to *Rht-B1* and *Rht-D1* semi-dwarfing genes. At the *Rht-B1* locus, the *Rht-B1b* allele for reduced height carried by Waagan was associated with 10.4 cm shorter plant height, and 18% decreased grain shattering, whereas *Rht-D1b* carried by Drysdale reduced plant height by 11.4 cm and reduced grain shattering by 20% (Fig. 6). These QTL were still significant, even after adjusting for the effect of plant height, but the explained variation was substantially reduced from 27.4 to 13.9% in the case of the *Rht-B1* locus, and from 18.9 to 6.7% for the *Rht-D1* locus. These results indicated a pleiotropic influence of the plant height major genes on grain shattering in the population.

**Table 3:** Main-effect quantitative trait loci (QTL) associated with grain shattering in two bi-parent populations of wheat

Marker	Chr	Pos. (Mb)	Effect	Prob	%Var	ANOVA test	
						Marker	M × E
Drysdale- × -Waagan							
IWA3730	3A	53.19	0.25	< 0.001	6.80	0.012	0.015
*Rht-B1*	4B	30.86	0.53	< 0.001	27.40	< 0.001	< 0.001
*Rht-D1*	4D	18.78	− 0.42	< 0.001	18.90	< 0.001	< 0.001
IWA6434	6A	594.83	0.17	0.002	3.30	0.010	0.061
IWA4486	6B	519.15	− 0.18	0.003	3.90	0.005	0.005
IWA418	7B	97.31	− 0.20	0.001	4.30	< 0.001	0.001
Crusader-×-RT812							
1248362	2A	74.25	− 0.14	0.001	4.30	< 0.001	0.56
1144438	2A	692.73	− 0.11	0.006	3.00	< 0.001	0.20
3945645	2B	663.13	0.18	< 0.001	7.00	< 0.001	0.33
4329714	2D	586.94	0.13	0.001	3.70	< 0.001	< 0.001
1127861	3A	718.34	0.27	< 0.001	7.80	0.01	0.71
2244885	3D	457.84	0.17	< 0.001	5.90	< 0.001	0.78
1048025	4A	330.09	− 0.17	0.001	3.30	< 0.001	0.53
1107268	5A	705.90	− 0.26	< 0.001	15.20	< 0.001	0.32
2322338	7B	712.83	0.13	0.001	4.20	< 0.001	0.54
1105401	7D	65.94	− 0.14	< 0.001	4.70	< 0.001	0.28

There were four minor QTL, located on chromosomes 3A, 6A, 6B and 7B (Fig. 5; Table 3). The minor QTL on 3A and 7B shared co-location with QTL associated with other agronomic traits, but the loci on chromosomes 6A and 6B were independent (Fig. 5a). Indeed, the locus on chromosome 3A was no longer significant, after adjusting for plant height, indicating it was a pleiotropic effect. However, the two loci detected on chromosome 6A and 6B, along with the QTL on chromosome 7B were still significant, even after conditioning on plant height, indicating they represent independent QTL. A QTL was detected for plant height on the short arm of chromosome 6A, which did not appear to affect shattering propensity. All identified QTL had some level of environmental sensitivity (Table 3), but this involved a change in magnitude rather than a change in direction.

### *QTL detected in Crusader* × *RT812*

Amongst parent of this mapping population, RT812 was shatter resistant, while Crusader was susceptible. As shown in Table 1, the doubled haploid progeny from the cross exhibited significant variability for grain shattering, with heritability that ranged from 0.42 to 0.87, depending on the environment. To investigate the genetic basis, the DNA of the progeny lines were assayed for polymorphism at DArTseq markers, and from a total of 9792, a subset of 3948 non-redundant markers were used for QTL analysis. These covered 15.7 Gigabase (Gb) of the 17-Gb hexaploid bread wheat genome (92.4%), with a density of one marker per 4 Mb. The markers, on average, satisfied the expected ratio of 1:1 (AA = 48.9%; BB = 51.1%).

Ten QTL were detected for grain shattering using the WGAIM algorithm, and the QTL × environment ANOVA testing showed the effects were largely stable across environments (Table 3). Half of the identified QTL (located on chromosomes 2A, 2B, 5A and 7D) were closely linked to QTL affecting other agronomic traits, while the other half were largely isolated (Fig. 5b). The major locus for grain shattering was detected on the long arm of chromosome 5A, approximately 433 Mb from the centromere. Based on in silico mapping against the reference wheat genome, this QTL was located about 54.1 Mb downstream of the wheat domestication gene, *Q,* which determines spike morphology. Support interval for the QTL was small, spanning 374.3 Kb in length, and contained six genes, including genes that encodes for Cytochrome P450 and UDP-glucosyltransferase.

The QTL with minor effects explained between 3 and 7.8% of the phenotypic variability, and the ANOVA test for main effects confirmed that all were significantly associated with grain shattering, and stable across environments. Two loci located at the apical and centromeric ends of chromosome 4A were no longer significant after adjusting for the plant height, despite there being no QTL for plant height at these regions. Apart from these two, all other QTL identified in the population were non-pleiotropic, independent genetic factors, as they were still significant after removing the influence of plant height.

## Discussion

In this study, a major locus was identified for grain shattering in the diversity panel, which mapped close to the *Tenacious glume* gene, *Tg*, on chromosome 2D. This locus was not evident in either of the bi-parent populations. This result is one of the benefits of using a combined linkage-based QTL mapping and LD-based association mapping to dissect the genetics of complex traits, as more genes can be identified by the combined use of these two methods (Lou et al. 2015). However, results obtained from such a combined approach are often not identical (Famoso et al. 2011; Lou et al. 2015; Altendorf et al. 2021), just as results from different bi-parent populations could be different due to heterogeneous genetic backgrounds (Holland 2007). In this case, one explanation is that the chromosome 2DS locus is probably fixed in the bi-parent populations. Parents of the doubled haploid populations were elite, high-yielding lines and not selected because of their contrasting phenotypes. QTL identified would be limited to the genetic loci segregating in the cross (Honsdorf et al. 2010; Brachi et al. 2010). The diversity panel, on the other hand, was comprised of old and current varieties, and the higher number of recombination events in such materials increase the range of natural variation that can be surveyed and would increase QTL detection power and resolution (Ewens and Spielman 2001).

Grain shattering is a difficult trait to phenotype accurately under field conditions (Zhang and Mergoum 2007a; Zhang et al. 2009), and in the past, breeding (Haley et al. 2005) and genetic studies (Marza et al. 2006; Bokore et al. 2022) have used visual scoring methods that are applied at harvest maturity. We followed the same approach here, and in a diverse panel of wheat cultivars, which comprised of elite lines and landrace varieties, this provided a good indication of a genotype’s propensity to shatter, with good stability across environments (Fig. 3). Substantial genetic variation was found to exist, with Spearman’s rank correlations ranging from 0.67 to 0.73, depending on the environment (Fig. 3). This meant that some wheat cultivars were more shatter resistant than others, and interestingly, more grain shattering occurred when crops were sown early (average score 4.3) than when sown late (average score 1.7). This has implications for early sowing systems to boost wheat yields (Hunt et al. 2019) because in bad years, a good portion of the harvest could be lost due to shattering.

Most of the shattering we observed in the field were at the apical end of the spike, that is, the top one-third of the spike (Fig. 1). This supports the findings made over 8 decades ago (Vogel 1941) that florets in the upper spikelets tend to shatter more easily than those towards the base of the spike. It could be that grains at the apical spikelets dry out faster during senescence and, therefore, more susceptible to be removable from the spike by buffeting wind and/or birds, but the physiological basis needs to be explored further.

We explored the genetic basis of grain shattering by QTL mapping in populations of different genetic backgrounds and to account for possible pleiotropy, we included other plant traits, such as phenology, plant height and grain size as covariates. The estimates of broad-sense heritability calculated for grain shattering in each population compared well with those calculated for plant height and phenology, suggesting it is either highly heritable on its own or it is the pleiotropic influence of genes affecting these traits. In the diversity panel, genome-wide analysis accurately identified the association of *Rht-B1* and *Rht-D1* with plant height, and these were the only markers above the threshold (Fig. 4), indicating presence of the *Rht* genes in the population. But there was no significant association of the *Rht* genes with grain shattering. Rather, the GWA identified a single locus, which explained 50% of the phenotypic variation in grain shattering. This was mapped to chromosome 2B (Wang et al. 2014), but the physical location was established to be the short arm of chromosome 2D, based on the updated Chinese Spring genome (RefSeq v2.1). Other previously reported genes on 2DS located in this vicinity include *Ppd-D1* and *Rht8*, based on sequence matches of closely linked markers (Jantasuriyarat et al. 2004). However, the most likely candidate gene is the *Tenacious glume, Tg1,* which is located ~ 10.0 Mb on the distal side, based on sequence matches of closely linked markers reported by Sood et al. (2009). The *Tg* genes governs the free-threshing habit in wheat (Kerber and Rowland 1974) and was mapped to short arms of chromosomes 2B and 2D by both Jantasuriyarat et al. (2004) and Sood et al. (2009). Until recently, most of the molecular markers for the genes were based on simple sequence repeats (SSR), but Arif et al. (2021) reported a SNP marker, wsnp_Ra_c25656_3522705, close to *Tg* gene on chromosome 2D. Its physical position on the Chinese Spring genome suggests it is co-located (~ 7.7 Mb) with the marker identified in this study.

### Grain shattering may be due to pleiotropic gene action

The genetic relationship of plant height with grain shattering is poorly understood, and some studies have suggested it might be entirely environmental (Zhang et al. 2009). Clarke and De Pauw (1983) found the amount of shattering was positively related with plant height, although not significant, and suggested that tall-strawed lines might tend to shatter more easily than short-strawed lines due to greater exposure of the spikes. In the Drysdale × Waagan cross, half of the QTL detected for grain shattering co-located with QTL associated with plant height and/or grain size (Fig. 5a). The two semi-dwarfing genes, *Rht-B1* and *Rht-D1*, accounted for most of the observed variation in grain shattering, with alleles at the *Rht-B1* locus contributing the most (*R*^2^ = 27.4%), and alleles at *Rht-D1* accounting for a large proportion (18.9%) of the observed variability. The *Rht-B1* locus explained 37.1% of the variation in plant height and 10.8% of the variability in grain size. On the other hand, the *Rht-D1* locus explained 43.2% of variation in plant height and only a small proportion (3.4%) of the variability in grain size.

It is conceivable, therefore, that the effect of these regions on grain shattering might be a pleiotropic action of the reduced height genes. The *Rht* genes encode mutant DELLA proteins that are negative regulators of several gibberellic acid responses required for growth and have been associated with some undesirable agronomic characteristics, especially in water-limited environments (Jatayev et al. 2020). A linkage of grain shattering with semi-dwarfism was suggested by Oba et al. (1990), and later, Nakamura et al. (1995) showed that grain shattering caused by a single recessive gene, *sh-2*, is linked to the well-known semi-dwarf gene, *sd-1* locus on chromosome 1. Zhang et al. (2013) reported the cloning of *TaqSH1*, a wheat ortholog of the rice grain shattering gene, *qSH1*, whose over-expression in *Arabidopsis thaliana* down-regulated known abscission genes and resulted in dwarfed plants, linking the reduced propensity to grain shattering with plant height.

The locus detected on chromosome 3A in the Drysdale × Waagan cross was no longer significant after adjusting for plant height. A significant QTL for plant height was detected within the interval, and this might be related to a gene for plant height identified by Martinez et al. (2021). QTL were also detected for grain shattering at the apical and centromeric ends of chromosome 4A in the Crusader × RT812 cross (Fig. 5b), but these became non-significant after adjusting for the plant height. This would indicate that these loci were associated with plant height and causally linked to grain shattering through the effect of plant height. Inability to detect a QTL for plant height at these regions might be related to allele frequency and small size of the effect (Würschum et al. 2017).

### Independent QTL for grain shattering

In the two bi-parent populations, we found significant QTL on multiple chromosomal regions other than those harbouring known *Rht* genes. The *Q* gene (TRAESCS5A02G473800), located on the long arm of chromosome 5A, controls the ease with which grains can be separated from the chaff (Simons et al. 2006). We did not detect the presence of the *Q* gene in either of the two bi-parent populations used for this study. In fact, no QTLs were detected on chromosome 5A in the Drysdale × Waagan cross. In the Crusader × RT812 population, a QTL with strong phenotypic effect was identified on chromosome 5AL, with the peak signal at marker, 1107268 (Table 3), but it is unlikely to be linked to the *Q* gene locus, as it is physically located about 55.8 Mb distal of *Q* gene location. Other previously reported QTL in this vicinity include loci identified by Jantasuriyarat et al. (2004) and more recently by Bokore et al. (2022).

We found multiple QTLs on homoeologous group 2 chromosomes in the Crusader × RT812 population (Fig. 5b; Table 3), and this was intriguing because genes controlling classic domestication traits are located on these chromosomes. The spike-compacting gene, *compactum* (*C*), which affects rachilla morphology and grain size, is located on long arm of chromosome 2D, in a segment near the centromere (Johnson et al. 2008). The marker for QTL detected on chromosome 2D physically mapped to the long arm of the chromosome, closer to the telomere than the centromere, and is therefore unlinked. Peleg et al. (2011) identified a gene for brittle rachis in a durum wheat × wild emmer population, which mapped to the long arm of chromosome 2A, but the QTL detected in the current study on the long arm of chromosome 2A (Fig. 5b; Table 3) mapped to approximately 41.2 Mb from this gene, and they are therefore unlikely to be linked.

In conclusion, our results suggest that grain shattering in standing crop of modern wheat is not related to any lingering presence of classical domestication genes. Rather, in a population segregating for the semi-dwarf genes, strong genetic linkage to the known *Rht* genes, *Rht-B1* and *Rht-D1*, was found, which supports suggestions in the literature of an association with plant height. The genetic correlation of grain shattering with plant height was strong and positive in the diversity panel, like in the Drysdale × Waagan bi-parent population (Table 2). Unlike the bi-parent population, however, there GWA did not find an association with *Rht* genes in the diversity panel. In fact, the landrace cultivars in the wheat diversity panel were taller than the elite cultivars by an average of 18.1 cm, but they were also the less likely to shatter. Single-marker analysis of variance showed that, to varying degrees of error, allelic variation at both *Rht-B1* (*P* = 0.002) and *Rht-D1* (*P* = 0.08) were associated with grain shattering in the diversity panel. Failure of GWA to detect an association might be due to stringency and can be regarded as a case of false negative.

QTL for grain shattering have been mapped to the *Rht* gene positions in previous studies (Marza et al. 2006; Bokore et al. 2022), but the current study is the first to use gene-based markers to confirm the association. The level of resolution is limited in these studies, and it would be worthwhile to further decipher the association of grain shattering with plant height and establish whether it is entirely pleiotropic or due to linkage disequilibrium. A SNP marker close to *Tenacious glume* gene, *Tg*, on chromosome 2DS was identified in the diversity panel and should be optimised for utility in marker-assisted selection.

## Supplementary Information

Below is the link to the electronic supplementary material.

Supplementary file1 (DOCX 67 kb)

## Data Availability

Data will be made available on request.
