# Supplementary material for: Natural variation and genetic loci underlying resistance to grain shattering in standing crop of modern wheat
Source: Mol Genet Genomics. 2023 Jul 6;298(5):1211–24. doi: 10.1007/s00438-023-02051-z (PMC10363068; doi:10.1007/s00438-023-02051-z)
Supplement: Supplementary file 1 — Supplementary file1 (DOCX 67 kb) [file 438_2023_2051_MOESM1_ESM.docx]

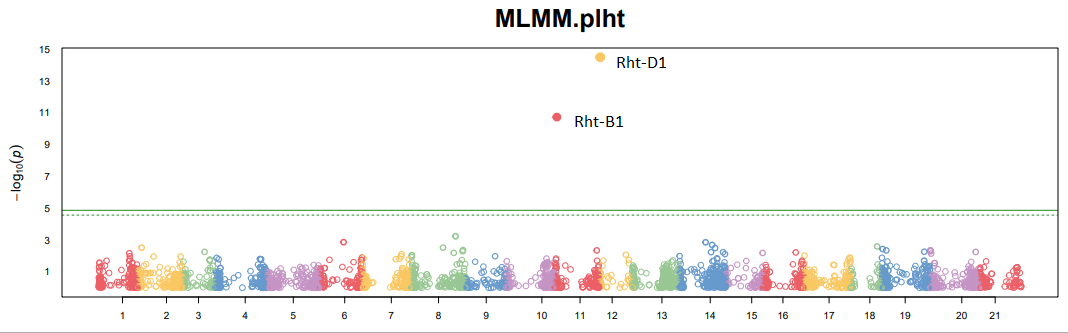


Supplementary Fig. 1. Manhattan plot from GAPIT analysis of plant height variation in the wheat diversity panel.
